# Hyperthermia in Human Ischemic and Hemorrhagic Stroke: Similar Outcome, Different Mechanisms

**DOI:** 10.1371/journal.pone.0078429

**Published:** 2013-11-04

**Authors:** Francisco Campos, Tomás Sobrino, Alba Vieites-Prado, María Pérez-Mato, Manuel Rodríguez-Yáñez, Miguel Blanco, José Castillo

**Affiliations:** 1 Department of Neurology, Neurovascular Area, Clinical Neurosciences Research Laboratory, Hospital Clínico Universitario, Health Research Institute of Santiago de Compostela (IDIS), University of Santiago de Compostela, Santiago de Compostela, Spain; St Michael's Hospital, University of Toronto, Canada

## Abstract

Hyperthermia is a predictor of poor outcome in ischemic (IS) and intracerebral hemorrhagic (ICH) stroke. Our aim was to study the plausible mechanisms involved in the poor outcome associated to hyperthermia in stroke. We conducted a case-control study including patients with IS (n = 100) and ICH (n = 100) within the first 12 hours from symptom onset. Specifically, IS and ICH patients were consecutively included into 2 subgroups, according to the highest body temperature within the first 24 hours: Tmax <37.5°C and Tmax ≥37.5°C, up to reach 50 patients per subgroup of temperature for both IS and ICH patients. Body temperature was determined at admission and every 4 hours during the first 48 hours. Main outcome variable was poor functional outcome (modified Rankin scale score >2) at 3 months. Serum levels of glutamate and active MMP-9 were measured at admission. Our results showed that Tmax ≥37.5°C within the first 24 hours was independently associated with poor outcome in both IS (OR, 12.43; 95% CI, 3.73–41.48; p<0.0001) and ICH (OR, 4.29; 95% CI, 1.32–13.91; p = 0.015) after adjusting for variables with a proven biological relevance for outcome. However, when molecular markers levels were included in the logistic regression model, we observed that glutamate (OR, 1.01; 95% CI, 1.00–1.02; p = 0.001) and infarct volume (OR, 1.06; 95% CI, 1.01–1.10; p = 0.015) were the only variables independently associated to poor outcome in IS, and active MMP-9 (OR, 1.04; 95% CI, 1.00–1.08; p = 0.002) and National Institute of Health Stroke Scale (NIHSS) at admission (OR, 1.29; 95% CI, 1.13–1.49; p<0.0001) in ICH. In conclusion, these results suggest that although the outcome associated to hyperthermia is similar in human IS and ICH, the underlying mechanisms may be different.

## Introduction

Demographic changes and improvement in quality of health care systems in developed countries are conditioning an increase in the incidence and prevalence of ischemic stroke (IS) and intracerebral hemorrhage (ICH). Therefore understand the molecular mechanisms involved in both neurological pathologies in order to find new and more efficient treatments are highly demanded.

Pharmacological (intravenous or intra-arterial) or mechanical reperfusion therapies are the only approved treatment during the acute phase of IS; however this treatment it is difficult to apply in more than 10% of patients due to its short therapeutic window and secondary complications [1]. In case of ICH, no satisfactory pharmacological treatments have been developed against this disorder with high mortality and poor prognosis rates [2]. Standard management for ICH is principally supportive, including airway protection, maintenance of hemodynamic stability, and control of intracranial pressure. In addition, it is well described that extravasation of blood molecular mediators into brain parenchyma after ICH mediates hematoma growth, edema and cell death. Therefore, early administration of hemostatic agents, meticulous blood pressure control, early surgical evacuation, and catheter hematoma aspiration have also been tried without success to limit hematoma expansion [3].

In both IS and ICH, hyperthermia is a common complication that occurs in up to 30–40% of patients, and it is independently associated with poor outcome and increased mortality [4]–[9]. However, although the molecular mechanisms underlying the deleterious effect of hyperthermia in IS is reasonably well described in ICH is largely unknown. Previous studies by our group, performed in animal models of cerebral ischemia, have demonstrated that the deleterious effect of hyperthermia is mediated mainly through the increase of glutamate excitotoxicity, while the protective effect associated to hypothermic treatments is tightly associated with a reduction of glutamate release [10], being these data also in agreement with our previous clinical data [11]. On the other hand, glutamate release seems to act as an important secondary mechanism of injury following hematoma growth in ICH [2]. In this regard, it was demonstrated in animal models of ICH that a transient elevation of the extracellular concentration of glutamate in the perihematomal region appears after hematoma formation. Likewise, memantine treatment, a low-affinity blocker of the N-methyl-D-aspartate subtype of glutamate receptor–associated channels, reduced hemorrhage volume, apoptotic cell death, neutrophil infiltration, and the number of microglia/macrophages in the periphery of hematoma [12], [13]. However, it is still largely unknown if glutamate excitotoxicity plays a critical role in the effect of hyperthermia during ICH.

Due to the adverse effect of hyperthermia in both IS and ICH outcome, our aim was to study whether glutamate release acts as a key molecular mechanism involved in the poor outcome associated to hyperthermia in human IS and ICH.

## Methods

### Study population

This is a case-control study including patients with IS (n = 100) and ICH (n = 100) within the first 12 hours from symptom onset. Specifically, IS and ICH patients were consecutively included into 2 subgroups, according to the highest body temperature within the first 24 hours: Tmax <37.5°C and Tmax ≥37.5°C, up to reach 50 patients per subgroup of temperature for both IS and ICH patients. Recruitment period was from April 2009 to July 2012. The study was carried out in accordance with the Declaration of Helsinki of the World Medical Association (2008) and approved by the Ethics Committee of Clinical Research of Galicia (CEIC). Written informed consent was obtained from each patient or their relatives after full explanation of the procedures.

According to the classification used in previous studies [14], [15], admission axillary temperature ≥37.5°C was considered as hyperthermia (independently of fever), whereas axillary temperature <37.5°C was considered as normothermia.

Following the clinical protocol of the stroke unit of our hospital, patients with axillary temperature ≥37.5°C were treated with metamizol (2 g intravenous) or paracetamol (500 mg orally) every 6 hours (although treatment with metamizol and paracetamol were used to control hyperthermia, the condition of hypothermia was not induced in any of the patients recruited).

Sample size for the present study was calculated using the statistical EPIDAT software (y, based on a prevalence of poor outcome >35% in stroke patients with hyperthermia according to previous studies [9], [15]. The minimum sample size calculated to detect this effect was made accepting an alpha level of 5% and a power of 80%.

This research was carried out in accordance with the Declaration of Helsinki of the World Medical Association (2008) and approved by the Ethics Committee of Clinical Research of Galicia (CEIC). Informed consent was obtained from each patient or their relatives after full explanation of the procedures.

### Clinical and neuroimaging variables

All patients were admitted in the acute stroke unit and handled by the same stroke team according to the Stroke Unit protocol of the Hospital Clínico Universitario de Santiago de Compostela.

Medical history recording potential vascular risk factors, blood and coagulation tests, 12-lead ECG, chest x-ray, and carotid ultrasonography were performed at admission.

In accordance with previous clinical studies [7] and following the Stroke Unit protocol of our hospital, axillary temperatures were measured by nurses. Temperature was obtained at admission and every 4 hours during the first 48 hours. Basal temperature and the highest temperature within the first 24 were considered for the analysis.

Stroke subtype was classified according to the TOAST criteria [16] in IS and as hypertensive, amyloid, in relation to antiplatelet/anticoagulant treatment, and secondary to arteriovenous malformation or others in ICH.

Stroke severity was assessed by an internationally certified neurologist using the National Institute of Health Stroke Scale (NIHSS) at admission, 24, 48 and 72 hours. Early neurological deterioration (END) was defined as an increase ≥4 points in NIHSS within the first 72 hours with respect to baseline NIHSS score. Functional outcome was evaluated at 3 months and poor functional outcome, the main outcome variable of the study, was defined as a modified Rankin Scale (mRS) score >2.

The use of reperfusion therapy, the inclusion in clinical trials and the presence of infections during the first 72 hours were also considered for the analysis.

All patients underwent cranial computed tomography (CT) at admission as well as a control CT between days 4 and 7. In patients with IS, a multimodal MRI (magnetic resonance imaging) was also performed at admission. Lesion volumes were measured using ABC/2 method [17] in DWI-MRI and control CT in patients with IS, and in CT at admission and control CT in patients with ICH. Neuroradiologists blinded to clinical and analytical data performed all imaging studies.

### Laboratory tests

Blood samples, obtained from all patients at admission were collected in chemistry test tubes, centrifuged at 3000 g for 15 minutes, and immediately frozen and stored at −80°C. Serum levels of Glu were determined by high performance liquid chromatography (HPLC) analysis following a previously described method [18], while active matrix metalloprotease- 9 (MMP-9) levels (GE Healthcare - Amersham, Little Chalfont Buckinghamshire,UK) were measured using commercial ELISA kits following manufacturer instructions. The intra-assay and inter-assay coefficients of variation were 1.7% and 2.3% for Glu, and 3.6% and 6.6% for active MMP-9, respectively. Determinations were performed in an independent laboratory blinded to clinical data.

### Statistical Analysis

Results were expressed as percentages for categorical variables and as mean (SD) or median and range (25^th^ and 75^th^ percentiles) for the continuous variables depending on whether their distribution was normal or not. The Kolmogorov-Smirnov test was used for testing the normality of the distribution. Proportions were compared using the chi-square or Fisher test, while the continuous variables between groups were compared with the Student's *t* or the Mann-Whitney tests. Spearman's or Pearson's analyses were used for bivariate correlations. ANOVA was used for comparison among several quantitative variables. The influence of molecular marker levels and temperature on poor functional outcome at 3 months was assessed by logistic regression analysis after adjusting for those variables with a proven biological relevance for outcome to avoid the possibility of finding some spurious associations, and that the effect of some predictors on the endpoint was not masked by the effect of some confounders. Specifically, the preselected outcome confounders for IS were age, history of atrial fibrillation, NIHSS at admission, glucose levels and DWI volume at admission [7]; while age, previous anticoagulants, history of hypertension, NIHSS and ICH volume at admission were preselected for ICH [6]. Results were expressed as adjusted odds ratios (OR) with the corresponding 95% confidence intervals (95% CI). The results of the OR corresponding to each unit of the corresponding variable (for example, per unit of NIHSS, per mL of DWI volume, per ng of MMP-9, per µM of glutamate, etc.). The statistical analysis was conducted using SPSS 16.0 (SPSS Inc. Chicago, IL, USA).

## Results

**Table 1** shows clinical, biochemical and neuroimaging characteristics of IS and ICH patients classified according to the cutoff of point of Tmax <37.5°C or Tmax ≥37.5°C for the highest body temperature within the first 24 hours. All groups were comparable regarding clinical characteristics, pharmacological treatments and IS or ICH subtype. However, both IS and ICH patients with Tmax ≥37.5°C showed worse functional outcome at 3 months, larger infarct or ICH volumes at 4^th^-7^th^ day as well as larger infarct or ICH growth, and increased frequency of infections. Furthermore, IS patients with Tmax ≥37.5°C showed higher levels of glucose, fibrinogen, high-sensitivity C-reactive protein (hs-CRP) and leukocytes, while ICH patients with Tmax ≥37.5°C had increased frequency of END. Regarding molecular markers, IS patients with Tmax ≥37.5°C showed higher levels of Glu. By contrast, ICH patients with Tmax ≥37.5°C showed higher levels of active MMP-9. In fact, we found strong correlations between Glu levels at admission and the highest body temperature within the first 24 hours (r = 0.458, p<0.0001) for IS patients, and between active MMP-9 levels at admission and the highest body temperature within the first 24 hours (r = 0.654, p<0.0001) for ICH patients.

**Table 1:** Clinical, biochemical and neuroimaging characteristics of ischemic and hemorrhagic stroke patients according to the cutoff of point of <37.5°C or ≥37.5°C for the highest body temperature within the first 24 hours.

VARIABLES	ISCHEMIC STROKE			INTRACEREBRAL HEMORRHAGE		
	<37.5°C	≥37.5°C	p	<37.5°C	≥37.5°C	p
	n = 50	n = 50		n = 50	n = 50	
**Age, years**	71.9±12.9	74.3±11.7	0.412	70.5±11.6	73.2±11.9	0.133
**Female, %**	37.0	52.0	0.061	48.0	38.0	0.419
**History of hypertension, %**	64.0	66.0	0.841	60.0	68.0	0.546
**History of diabetes, %**	20.0	12.0	0.414	6.0	20.0	0.071
**History of dyslipemia, %**	18.0	14.0	0.786	30.0	20.0	0.356
**Alcohol consumption, %**	8.0	12.0	0.741	12.0	16.0	0.774
**Smoking habit, %**	10.0	20.0	0.262	2.0	4.0	0.554
**History of peripheral vascular disease, %**	2.0	8.0	0.362	0	6.0	0.242
**History of ischemic heart disease, %**	10.0	6.0	0.715	4.0	12.0	0.269
**History of atrial fibrillation, %**	14.0	22.0	0.436	10.0	12.0	0.749
**History of heart failure, %**	4.0	8.0	0.678	2.0	2.0	1.000
**History of transient ischemic attack, %**	4.0	2.0	0.558	0	0	-
**Previous antiplatelets, %**	30.0	16.0	0.153	20.0	12.0	0.414
**Previous anticoagulants, %**	2.0	14.0	0.059	6.0	4.0	0.646
**Body temperature at admission,**°**C**	36.3±0.4	36.5±0.5	0.092	36.5±0.4	36.7±0.9	0.115
**Highest temperature within first 24 h,**°**C**	36.4±0.5	37.9±0.2	<0.0001	36.4±0.5	37.9±0.4	<0.0001
**Baseline systolic blood pressure, mm Hg**	153.4±27.5	150.6±32.2	0.836	158.7±25.7	157.3±32.9	0.123
**Baseline diastolic blood pressure, mm Hg**	83.9±15.9	81.6±18.7	0.475	86.2±16.4	80.7±16.8	0.877
**Glucose levels, mg/dL**	128.6±52.7	154.1±71.4	0.015	124.1±28.4	141.1±49.7	0.070
**Leukocytes, x10^3^/mL**	7.9±2.9	11.5±3.3	<0.0001	8.7±2.5	9.5±3.5	0.102
**Fibrinogen, mg/dL**	456.5±122.6	562.6±133.2	<0.0001	480.4±100.4	516.9±127.8	0.145
**hs-CRP, mg/L**	2.1±3.2	10.3±10.7	<0.0001	5.2±6.4	6.8±7.9	0.141
**NIHSS at admission**	9 [6], [15]	11 [9], [18]	0.069	9 [6], [16]	12 [7], [15]	0.111
**Early neurological deterioration (END), %**	2.0	10.0	0.092	2.0	26.0	<0.0001
**Infections during the first 72 hours, %**	2.0	34.0	<0.0001	2.0	30.0	<0.0001
**Ischemic stroke subtype**			0.136			-
** - Atherothrombotic, %**	26.0	28.0		-	-	
** - Cardioembolic, %**	34.0	44.0		-	-	
** - Lacunar, %**	10.0	10.0		-	-	
** - Undetermined, %**	30.0	18.0		-	-	
**Intracerebral hemorrhage subtype**			-			0.123
** - Hypertensive, %**	-	-		40.0	32.0	
** - Amyloid, %**	-	-		22.0	26.0	
** - Antiplatelet/anticoagulant treatment, %**	-	-		14.0	24.0	
** - Other, %**	-	-		24.0	18.0	
**Thrombolytic treatment, %**	8.0	8.0	1.000	-	-	-
**Inclusion in clinical trial, %**	6.0	6.0	1.000	0	0	-
**DWI volume at admission, mL**	41.4±30.3	56.3±54.8	0.553	-	-	-
**Infarct volume at 4^th^-7^th^ day, mL**	16.8±31.9	55.4±57.6	<0.0001	-	-	-
**Infarct volume growth, %**	-72.8±23.5	-3.6±16.6	<0.0001	-	-	-
**ICH volume at admission, mL**	-	-	-	34.1±21.7	51.9±56.9	0.200
**ICH volume at 4^th^-7^th^ day, mL**	-	-	-	27.6±22.4	68.5±67.8	<0.0001
**ICH growth, %**	-	-	-	-22.5±23.0	52.3±80.2	<0.0001
**Glutamate levels at admission, µM**	118.5±106.7	226.2±97.3	<0.0001	121.8±86.9	137.6±96.7	0.385
**Active MMP-9 at admission, ng/mL**	27.6±12.3	28.8±13.7	0.620	17.3±9.4	48.2±14.4	<0.0001
**Modified Rankin Scale score at 3 months**	1 [0, 3]	4 [3], [6]	<0.0001	2 [0, 3]	3 [2], [6]	<0.0001

In the multivariate analysis, Tmax ≥37.5°C within the first 24 hours was independently associated with poor outcome in both IS (OR, 12.43; 95% CI, 3.73-41.48; p<0.0001) (**Table 2**) and ICH (OR, 4.29; 95% CI, 1.32-13.91; p = 0.015) (**Table 3**) patients after adjusting for variables with a proven biological relevance for outcome. However, when molecular markers levels were included in the logistic regression model, we observed that glutamate (OR, 1.01; 95% CI, 1.00-1.02; p = 0.001) and infarct volume (OR, 1.06; 95% CI, 1.01-1.10; p = 0.015) were the only variables independently associated to poor outcome in IS, and active MMP-9 (OR, 1.04; 95% CI, 1.00-1.08; p = 0.002) and NIHSS at admission (OR, 1.29; 95% CI, 1.13-1.49; p<0.0001) in ICH (**Table 2** **and** **3**).

**Table 2:** Adjusted OR of the Tmax ≥37.5°C within the first 24 hours and molecular markers levels for poor functional outcome at 3 months in ischemic stroke.

Independent variable	Non-adjusted		Adjusted	
	OR (95% CI)	p	OR (95% CI)	p
Model without molecular markers				
**Age**	1.02 (0.99 – 1.06)	0.154	1.00 (0.96 – 1.05)	0.912
**History of atrial fibrillation**	1.17 (0.41 – 3.32)	0.768	1.12 (0.26 – 5.37)	0.831
**NIHSS at admission**	1.16 (1.08 – 1.25)	<0.0001	1.07 (0.97 – 1.18)	0.150
**Glucose levels at admission**	1.00 (0.99 – 1.01)	0.316	1.00 (0.99 – 1.01)	0.903
**DWI volume at admission**	1.04 (1.01 – 1.06)	0.003	1.04 (1.00 – 1.08)	0.037
**Tmax** ≥**37.5°C within first 24 h**	11.15 (4.26 – 29.18)	<0.0001	12.43 (3.73 – 41.48)	<0.0001
Model with molecular markers				
**Age**	1.02 (0.99 – 1.06)	0.154	1.01 (0.95 – 1.07)	0.732
**History of atrial fibrillation**	1.17 (0.41 – 3.32)	0.768	2.05 (0.30 – 13.82)	0.462
**NIHSS at admission**	1.16 (1.08 – 1.25)	<0.0001	1.06 (0.94 – 1.19)	0.327
**Glucose levels at admission**	1.00 (0.99 – 1.01)	0.316	1.00 (0.99 – 1.02)	0.300
**DWI volume at admission**	1.04 (1.01 – 1.06)	0.003	1.06 (1.01 – 1.10)	0.015
**Tmax** ≥**37.5°C within first 24 h**	11.15 (4.26 – 29.18)	<0.0001	4.95 (0.92 – 12.75)	0.061
**Glutamate levels**	1.01 (1.00 – 1.02)	<0.0001	1.01 (1.00 – 1.02)	0.001
**Active MMP-9 levels**	1.05 (1.02 – 1.09)	0.005	1.13 (0.94 – 1.23)	0.057

**Table 3:** Adjusted OR of the Tmax ≥37.5°C within the first 24 hours and molecular markers levels for poor functional outcome at 3 months in intracerebral hemorrhage.

Independent variable	Non-adjusted		Adjusted	
	OR (95% CI)	P	OR (95% CI)	p
Model without molecular markers				
**Age**	1.03 (0.99 – 1.06)	0.123	0.98 (0.94 – 1.03)	0.462
**Previous anticoagulants**	2.95 (0.47 – 18.58)	0.248	1.00 (0.11 – 9.49)	1.006
**History of hypertension**	1.54 (0.67 – 3.58)	0.312	1.35 (0.44 – 4.16)	0.599
**NIHSS at admission**	1.32 (1.18 – 1.47)	<0.0001	1.28 (1.13 – 1.45)	<0.0001
**ICH volume at admission**	1.03 (1.01 – 1.05)	0.004	1.01 (0.98 – 1.03)	0.533
**Tmax** ≥**37.5°C within first 24 h**	4.00 (1.65 – 9.71)	0.002	4.29 (1.32 – 13.91)	0.015
Model with molecular markers				
**Age**	1.03 (0.99 – 1.06)	0.123	0.98 (0.93 – 1.03)	0.532
**Previous anticoagulants**	2.95 (0.47 – 18.58)	0.248	1.37 (0.14 – 13.12)	0.785
**History of hypertension**	1.54 (0.67 – 3.58)	0.312	1.30 (0.40 – 4.21)	0.658
**NIHSS volume at admission**	1.32 (1.18 – 1.47)	<0.0001	1.29 (1.13 – 1.49)	<0.0001
**ICH volume at admission**	1.03 (1.01 – 1.05)	0.004	1.01 (0.98 – 1.03)	0.432
**Tmax** ≥**37.5°C within first 24 h**	4.00 (1.65 – 9.71)	0.002	1.73 (0.59 – 5.07)	0.314
**Glutamate levels**	1.00 (0.99 – 1.01)	0.276	0.99 (0.98 – 1.01)	0.423
**Active MMP-9 levels**	1.04 (1.01 – 1.07)	0.003	1.04 (1.00 – 1.08)	0.002

## Discussion

This study shows that body temperature within the first 24 hours ≥37.5°C predict poor outcome in both patients with IS and ICH. These results are in line with the well-established deleterious effect of hyperthermia in these neuronal pathologies [6], [7]. However, while IS patients with Tmax ≥37.5°C showed higher levels of glutamate, ICH patients with Tmax ≥37.5°C showed higher levels of active MMP-9. These clinical data seems to indicate that in IS the deleterious effect of hyperthermia on functional outcome could be mediated by glutamate and infarct volume, in ICH is mediated mainly by active MMP-9 and neurological deficit at admission. Therefore, although the poor outcome associated to hyperthermia is similar in patients with IS and ICH, the underlying mechanisms may be completely different.

The results of this study show the critical role of glutamate in the deleterious effect of hyperthermia during acute phase of IS. These findings support our previous experimental data, where we have showed that temperature effects are strongly associated to glutamate excitotoxicity. In fact, analysis of the inflammatory response and metabolic rate demonstrates that effects of hyperthermia on ischemic damage were also less critical than glutamate excitotoxity [10]. In line with our previous study [10], our findings strengthen the hypothesis that the beneficial effect of treatments focused in the reduction of hyperthermia after ischemia, such as hypothermia or antipyretic drugs, could be enhanced in combination with drugs able to reduce the glutamate excitotoxicity (see [19] for review).

On the other hand, it has been widely described the relationship between hyperthermia and poor functional outcome after ICH [9], [14], [20]. However, the molecular mechanisms associated to the deleterious effects of hyperthermia in ICH have not yet been fully clarified. It has been suggested that molecular processes such as inflammation, glutamate excitotoxicity, infections, and processes in relation to the hematoma growth, which induces early pathophysiologic changes in the surrounding brain tissue such as breakdown of the brain-blood barrier (BBB) and development of vasogenic edema, considered relevant predictors of poor outcome in ICH, could be involved in the deleterious consequences of hyperthermia [6], [20]. In our study, we found that the association between hyperthermia and poor outcome in ICH seems to be mainly mediated by active MMP-9 (a biomarker of BBB breakdown) and the baseline neurological deficit, but not by an increase of glutamate levels as occurs in IS. Hematoma growth is one of the main physiological complications associated to poor outcome in ICH [14], [20]. Although the precise mechanisms involved in the deleterious effect of early hematoma growth during the acute phase is also poorly understood, MMPs overexpression and breakdown of the BBB are proposed as two of the most important processes associated to hematoma growth [3]. Likewise, MMP-9 seems to be also involved in secondary brain injury and outcome after primary ICH in humans [21]–[23]. Therefore, the results of this study suggest for first time that, over other mechanisms, increase of active MMP-9 represents the one of the most critical mechanism involve in the deleterious effect of hypothermia in ICH. Therefore, it is tentative to postulate that the management of hyperthermia in ICH patients should include treatments able to reduce hematoma or MMP-9 activity. In this regard, the use of treatments with antipyretics and/or cooling blankets in combination with drugs against the hematoma extension could help to reduce the deleterious effect of hyperthermia.

Interestingly, our results showed that glutamate excitotoxicity does not seem to act as a critical mechanism involved in the deleterious effect of hyperthermia in ICH as happens with ischemia. Although experimental studies have demonstrated that glutamate is transiently accumulated in the perihematoma region during the early phase of ICH, the specific role of glutamate in the brain injury observed after ICH needs to be further explored. In this regard, in the current animal models (autologous blood or collagenase injection) of ICH it is difficult to know if glutamate increase is produced as result of the artificial effect of the tissue disruption after blood or collagenase injection, or it is due to the mass effect of perihematomal edema, which can cause a regional hypoperfusion by mechanical compression of blood vessels [24]. Moreover, clinical studies have not even demonstrated the relevance of glutamate in ICH. Therefore further experimental and clinical studies are necessary to elucidate the role of glutamate in ICH.

Due to hyperthermia is quite different from fever in several fundamental aspects, including hypothalamic set point change in fever but not in hyperthermia, we considered that one limitation of the study was that patients with hyperthermia were not classified in those with and without fever. Nevertheless, since body temperature is the main variable but not the physiological mechanism involved in the increase of temperature, we consider that this limitation does not affect to the conclusions of our results.

In conclusion, the present study demonstrates that body temperature within the first 24 hours ≥37.5°C predict poor outcome in both IS and ICH patients, but the underlying mechanisms are different, glutamate excitotoxicity and infarct volume in IS and active MMP-9 and neurological deficit in case of ICH. Therefore, future protective strategies focused on the management of hyperthermia effects in both IS and ICH should be design taking into account the mechanism involved.
